# Personalized treatment in HPV^+^ oropharynx cancer using genomic adjusted radiation dose

**DOI:** 10.1172/JCI194073

**Published:** 2025-09-25

**Authors:** Emily Ho, Loris De Cecco, Steven A. Eschrich, Stefano Cavalieri, Geoffrey Sedor, Frank Hoebers, Ruud H. Brakenhoff, Kathrin Scheckenbach, Tito Poli, Kailin Yang, Jessica A. Scarborough, Shivani Nellore, Shauna Campbell, Neil Woody, Tim Chan, Jacob Miller, Natalie Silver, Shlomo Koyfman, James Bates, Jimmy J. Caudell, Michael W. Kattan, Lisa Licitra, Javier F. Torres-Roca, Jacob G. Scott

**Affiliations:** 1 Department of Genomic Medicine, Cleveland Clinic Research, Cleveland, Ohio, USA.; 2 School of Medicine, Case Western Reserve University, Cleveland, Ohio, USA.; 3 Integrated Biology of Rare Tumors, Experimental Oncology Department, Fondazione IRCCS Istituto Nazionale dei Tumori, Milan, Italy.; 4 Department of Biostatistics and Biomedical Informatics, Moffitt Cancer Center, Tampa, Florida, USA.; 5 Head and Neck Medical Oncology Department, Fondazione IRCCS Istituto Nazionale dei Tumori, Milan, Italy.; 6 Department of Oncology and Hemato-oncology, University of Milan, Milan, Italy.; 7 Radiation Oncology Department, NewYork-Presbyterian Hospital/Columbia University Vagelos College of Physicians and Surgeons, New York, New York, USA.; 8 Department of Radiation Oncology (Maastro), GROW School for Oncology and Reproduction, Maastricht University Medical Centre+, Maastricht, Netherlands.; 9 Amsterdam UMC, Vrije Universiteit Amsterdam, Otolaryngology/Head and Neck Surgery, Cancer Center Amsterdam, Amsterdam, Netherlands.; 10 Department of Otolaryngology, Medical Faculty, Heinrich Heine University Dusseldorf, Dusseldorf, Germany.; 11 Unit of Maxillofacial Surgery, Department of Medicine and Surgery, University of Parma–University Hospital of Parma, Parma, Italy.; 12 Department of Radiation Oncology, Holden Comprehensive Cancer Center, University of Iowa, Iowa City, Iowa, USA.; 13 Department of Radiation Oncology, and; 14 Center for Immunotherapy and Precision Immuno-Oncology, Cleveland Clinic, Cleveland, Ohio, USA.; 15 Department of Radiation Oncology, Emory University, Atlanta, Georgia, USA.; 16 Department of Radiation Oncology, Moffitt Cancer Center, Tampa, Florida, USA.; 17 Department of Quantitative Health Sciences, Cleveland Clinic, Cleveland Ohio, USA.; 18 Departments of Physics and Biology, Case Western Reserve University, Cleveland, Ohio, USA.

**Keywords:** Clinical Research, Oncology, Biomarkers, Head and neck cancer, Radiation therapy

## Abstract

**BACKGROUND:**

A key objective in managing HPV^+^ oropharyngeal squamous cell carcinoma (OPSCC) is reducing radiation therapy (RT) doses without compromising cure rates. A recent phase II/III HN005 trial revealed that clinical factors alone are insufficient to guide safe RT dose de-escalation. Our prior research demonstrated that the genomic adjusted radiation dose (GARD) predicts RT benefit and may inform dose selection. We hypothesize that GARD can guide personalized RT de-escalation in HPV^+^ OPSCC patients.

**METHODS:**

Gene expression profiles were analyzed in 191 HPV^+^ OPSCC patients enrolled in an international, multi-institutional observational study (AJCC Eighth Edition, stages I–III). Most patients received 70 Gy in 35 fractions or 69.96 Gy in 33 fractions (median dose: 70 Gy; range: 51.0–74.0 Gy). Overall survival (OS) was 94.1% at 36 months and 87.3% at 60 months. A Cox proportional hazards model assessed association between GARD and OS, and time-dependent receiver operating characteristic analyses compared GARD with traditional clinical predictors.

**RESULTS:**

Despite uniform RT dosing, GARD showed wide heterogeneity, ranging from 15.4 to 71.7. Higher GARD values were significantly associated with improved OS in univariate (HR = 0.941, *P* = 0.041) and multivariable analyses (HR = 0.943, *P* = 0.046), while T and N stages were not. GARD demonstrated superior predictive performance at 36 months (AUC = 78.26) versus clinical variables (AUC = 71.20). Two GARD-based RT de-escalation strategies were identified, offering potential survival benefits while reducing radiation exposure.

**CONCLUSION:**

GARD predicts OS and outperforms clinical variables, supporting its integration into the diagnostic workflow for personalized RT in HPV^+^ OPSCC.

**FUNDING:**

This work was supported by the National Cancer Institute through the Cleveland Clinic/Emory ROBIN center (U54-CA274513, project 2), the European Union Horizon 2020 Framework Programme (grant/award 689715), the Italian Association for Cancer Research (AIRC project ID 23573), and the European Research Area Network ERA PerMed JTC2019/Fondazione Regionale per la Ricerca Biomedica project SuPerTreat (Supporting Personalized Treatment Decisions in Head and Neck Cancer through Big Data).

## Abstract 2

Using a large trial of patients treated with radiation therapy for head and neck cancers we show the utility of a genomic method personalizing radiation dose for each patient to optimize their outcomes and minimize their toxicity.

## Graphical abstract

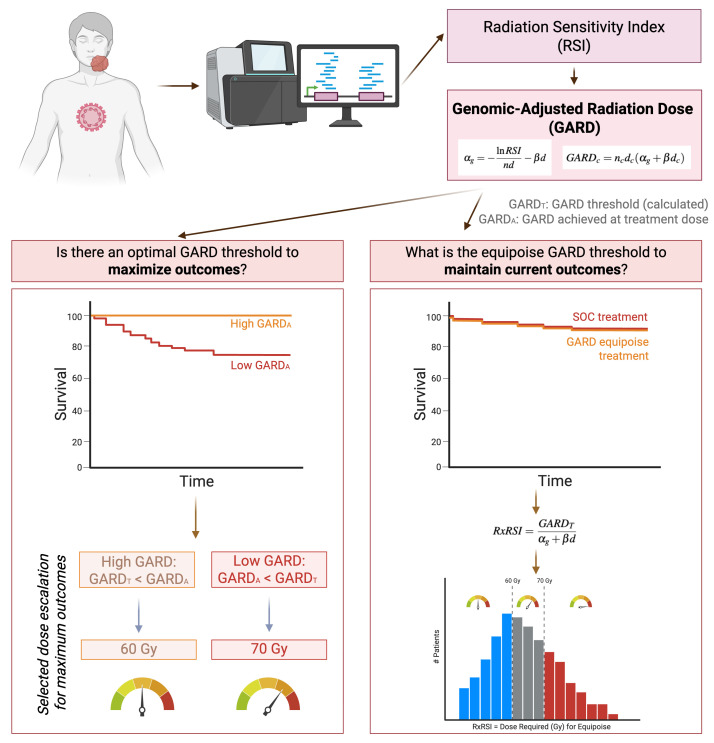

## Introduction

Since the discovery that HPV is an etiologic and strong prognostic factor in oropharyngeal squamous cell carcinoma (OPSCC), assessing this biomarker indirectly via p16 or directly via ISH has become the standard of care (SOC) in the diagnostic and staging workup of these patients (1, 2). Ang et al. developed a 3-group classification system based on clinical factors (HPV status, smoking pack years, and T or N classification) that has informed the design of multiple clinical trials in the last decade (3). As the low-risk group in this classification had an overall survival (OS) of 93% at 3 years, it was hypothesized that this favorable subset could be treated to a lower radiation therapy (RT) dose/toxicity without compromising OS. Thus, the development of a successful uniform approach to RT dose de-escalation has been a central clinical aim of the field over the last decade.

While several approaches to RT dose de-intensification have been explored, all of them share many characteristics. First, they define eligibility based on clinical factors that define prognostic risk. For example, the NRG Oncology focused on low-risk HPV^+^ patients receiving primary RT, while the Memorial Sloan Kettering Cancer Center (MSKCC) approach (4) focused on low-risk patients with a negative F-MISO PET scan. Second, they assume all patients are biologically homogeneous and have the same opportunity to benefit from RT. Therefore, the RT de-escalation strategy utilized is uniform. NRG chose 60 Gy with concurrent cisplatin, while MSKCC chose uniform 30 Gy to the neck after surgery with no chemotherapy.

Recently, the NRG announced early results of HN005, a phase II/III clinical trial testing the noninferiority of uniform RT dose de-escalation (cisplatin + 60 Gy or nivolumab + 60 Gy) against the SOC (cisplatin + 70 Gy) (5). Unfortunately, the phase II portion failed to demonstrate the noninferiority of cisplatin + 60 Gy over SOC. These early results suggested that clinical factors and a uniform therapeutic approach are not enough to provide therapeutic guidance for RT de-intensification and suggest that similar to many targeted (6) and immunotherapy agents (7), RT dose optimization may need to be targeted to specific genomically defined subpopulations.

In previous studies, we developed the genomic adjusted radiation dose (GARD), a radiation-specific metric that quantifies the RT treatment effect in a given patient as a function of their RT dose and tumor genomics (8). GARD results suggested that the treatment effect of a uniform dose of RT (e.g., 70 Gy) is biologically highly heterogeneous, rather than homogenous, the current assumption in the field. In a recent pooled analysis of 1,615 patients in 7 different disease sites, we demonstrated that GARD was associated with OS and recurrence risk as a continuous variable and predicted RT treatment benefit for each individual patient (9). Since GARD quantifies the treatment benefit for each patient, GARD-based models can be used to inform RT dose adjustments to optimize a patient’s clinical outcome, thus providing a tool to personalize RT dose.

GARD provides a critical innovation compared with the current approaches to RT de-intensification: disproving the assumption that RT benefit is homogenous and the limitation of uniform RT dosing strategies. However, the opportunity to improve the outcome of RT-treated patients is different in each disease. Therefore, it is critical that disease site–specific GARD models are developed. Since RT benefit is 1 of the critical factors defining clinical outcome in HPV^+^ head and neck squamous cell carcinoma (HNSCC) patients, we hypothesized that GARD could provide information that will allow for more personalized approaches to the successful treatment of these patients.

To test whether GARD could serve this purpose, we first assessed its prognostic ability in a modern cohort of radiation-treated patients with HPV^+^ HNSCC. Furthermore, we hypothesized that GARD’s prognostic information would provide an improvement to outcome prediction compared with clinical factors alone. Since GARD is intrinsically linked to radiation dose, any improvement in outcome prediction utilizing GARD can also fundamentally be used to make quantitative predictions for differential outcome given specific RT dose adjustments. This provides not just a tool for the definition of subpopulations for clinical trial design, but also the opportunity for truly personalized radiation dosing in the clinic.

Here, we describe an analysis of patients treated with RT with HPV^+^ HSNCC as part of the Big Data to Decide (BD2Decide) Project (10). We used modern methods to assess individual patient radiation sensitivity indices (RSIs) (11) from gene expression data derived from formalin-fixed tumor specimens and used radiation dosing information for each patient to calculate GARD. We then used continuous Cox proportional hazards regression to determine the relationship between GARD and outcome, and present a discrete analysis at several cut points post hoc to suggest optimal stratification strategies. Finally, we developed GARD-based models to personalize RT dose to achieve the best possible clinical outcome (both tumor and normal tissue) for each patient. We found that it is not only possible to reduce RT dose for a substantial number of patients but that it is also possible to improve tumor outcomes with RT dose personalization.

## Results

### HPV^+^ OPSCC cohort.

We identified 191 patient tumors previously profiled through the BD2Decide Project that met the criteria of definitive RT (12) (for details on patient selection, see Supplemental Figure 1; supplemental material available online with this article; https://doi.org/10.1172/JCI194073DS1). The characteristics for these HPV^+^ OPSCC patients are detailed in Supplemental Table 1.

### GARD reveals underlying genomic heterogeneity in RT effect.

**Figure 1:**
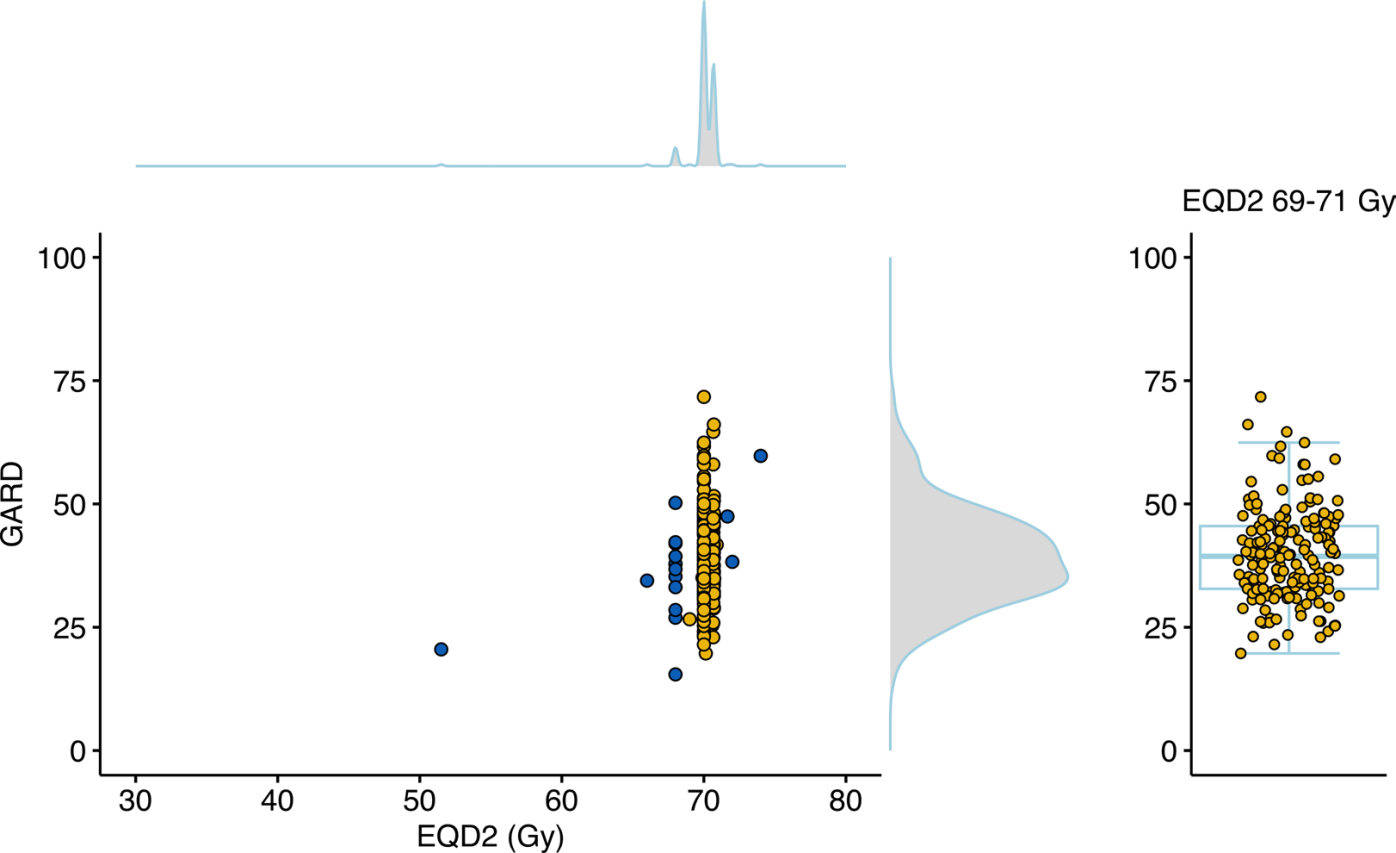
GARD exhibits large underlying genomic heterogeneity in radiation effect compared with radiation dose alone. Left: EQD2 (median = 70.0, IQR = 0.7) is plotted against associated GARD (median = 39.1, IQR = 12.6) for each patient in the cohort. Kernel density estimates are plotted on each edge to show the distributions of the individual variables. Patients that received an EQD2 of 69–71 Gy (standard dosing) are indicated in yellow. Right: The GARD value of all patients receiving an EQD2 of 69–71 Gy (SOC) highlight GARD’s ability to stratify patients by their genomic heterogeneity. Data points are overlaid with a box-and-whisker plot, with boxes representing quartiles and whiskers extending to 1.5 times IQR.

We have previously shown that GARD reveals underlying heterogeneity in radiation treatment effect within groups presumed to have been treated uniformly (with approximately equivalent physical dose) (8, 9, 13, 14). In this cohort, we demonstrate again that GARD reveals wide heterogeneity in predicted RT effect despite relatively uniform RT dose prescribed. As shown in Figure 1, delivered GARD ranged from 15.4 to 71.7 (median = 39.1, IQR = 12.6). Plotted along the edges of the joint plot between GARD and equivalent dose in 2 Gy fractions (EQD2) are kernel density estimates for the entire cohort, revealing wide heterogeneity in delivered GARD (IQR = 12.6) in the setting of near homogeneity in RT dose (IQR = 0.04). The difference between GARD and EQD2 is best exemplified by the patients who received the whole course of standard radiation dose, with EQD2 measures between 69 and 71 Gy (see Figure 1). The range of GARDs for those patients was 19.7–71.7 (IQR = 12.7) even though they all were treated to (approximately) the same RT dose (EQD2), highlighting the wide differential in the predicted effect of our uniform clinical dosing strategies. The distributions of RSI and GARD by AJCC stage (AJCC Eighth Edition) did not differ significantly (Supplemental Figure 2 and Supplemental Table 3).

### GARD is continuously associated with OS in RT-treated HPV^+^ OPSCC patients.

**Figure 2:**
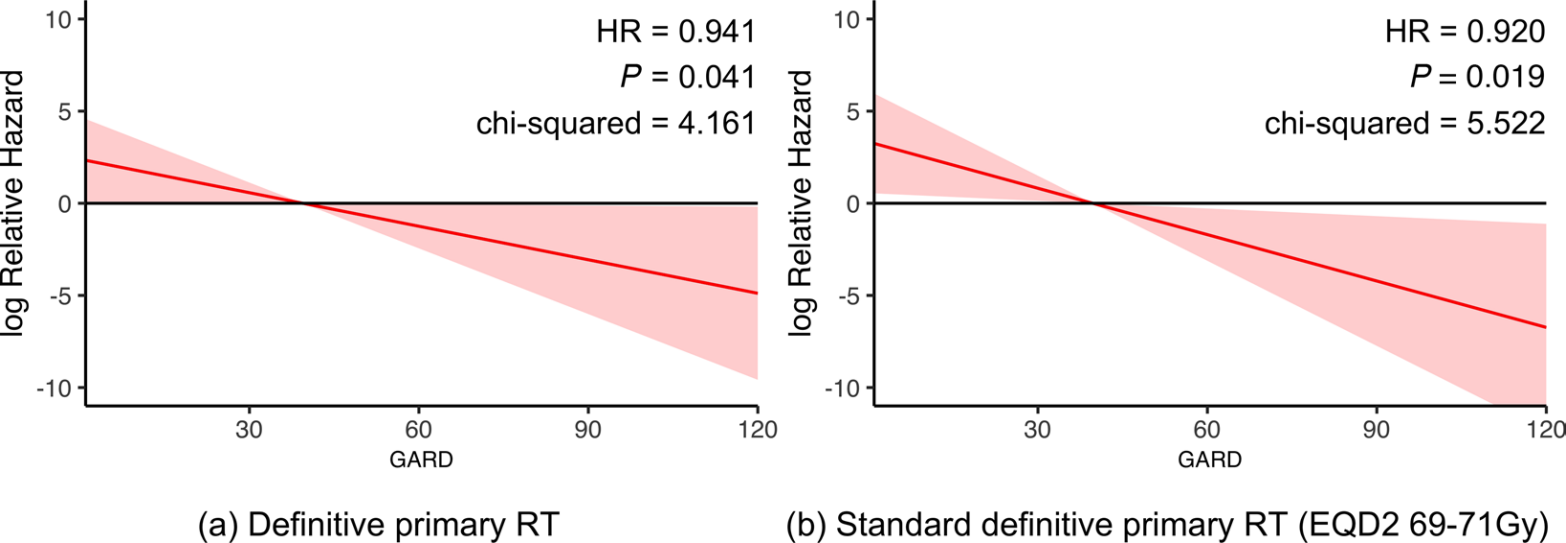
GARD is a continuous predictor of OS in radiation-treated patients with HPV^+^ OPSCC. (**A**) Cox proportional hazards analysis demonstrates significant continuous association between GARD and OS for patients treated with primary definitive RT and censored at 60 months [*P* = 0.041, HR = 0.941 (0.888, 0.998) per unit GARD]. (**B**) Cox proportional hazards analysis demonstrates significant continuous association between GARD and OS for the subset of patients treated with SOC primary definitive RT (EQD2, 69–71 Gy) [*P* = 0.019, HR = 0.920 (0.857, 0.986) per unit GARD].

Previously, we demonstrated that GARD was associated with OS and recurrence risk and was predictive of RT benefit in a pooled pan-cancer analysis of 1,615 patients, including cohorts with HSNCC (9). Since RT therapeutic benefit is a critical factor impacting clinical outcome in HPV^+^ patients, we hypothesized that GARD would be associated with clinical outcome in this analysis of HPV^+^ OPSCC patients collected through the BD2Decide Project (12). To test this hypothesis, we performed a Cox proportional hazards analysis of GARD and OS in patients that were treated with definitive primary RT (*n* = 191) and those treated with SOC definitive primary RT (EQD2, 69–71 Gy) (*n* = 174). As shown in Figure 2, GARD is associated with OS as a continuous variable for patients treated with primary definitive RT and censored at 60 months. We found that for each unit increase in GARD there was an improvement in OS [HR (95% CI) = 0.941 (0.888, 0.998) per unit GARD, *P* = 0.041]. This association of GARD with OS also held when including only patients treated with primary definitive RT at SOC doses (EQD2, 69–71 Gy), as shown in Figure 2 [HR (95% CI) = 0.920 (0.857, 0.986) per unit GARD, *P* = 0.019]. These findings suggest that GARD can stratify patients by predicted effect even when radiation dose is approximately uniform.

### GARD is associated with OS in RT-treated HPV^+^ OPSCC patients.

The BD2Decide cohort includes clinical variables for performance status (Eastern Cooperative Group [ECOG] > 0), T stage (T4 vs. T1–3), N stage (N2–N3 vs. N0–N1), and smoking pack years (>10). We performed univariable and multivariable analysis using these variables and GARD for statistical associations with OS. In the definitive primary RT cohort, N stage was significantly associated with OS in univariate analysis (*P* = 0.048), but in multivariate analysis, GARD was the only variable statistically associated with OS [HR = 0*.*943 (0*.*891, 0*.*999), *P* = 0*.*046]. These results are summarized in Table 1 and Supplemental Table 2.

**Figure 3:**
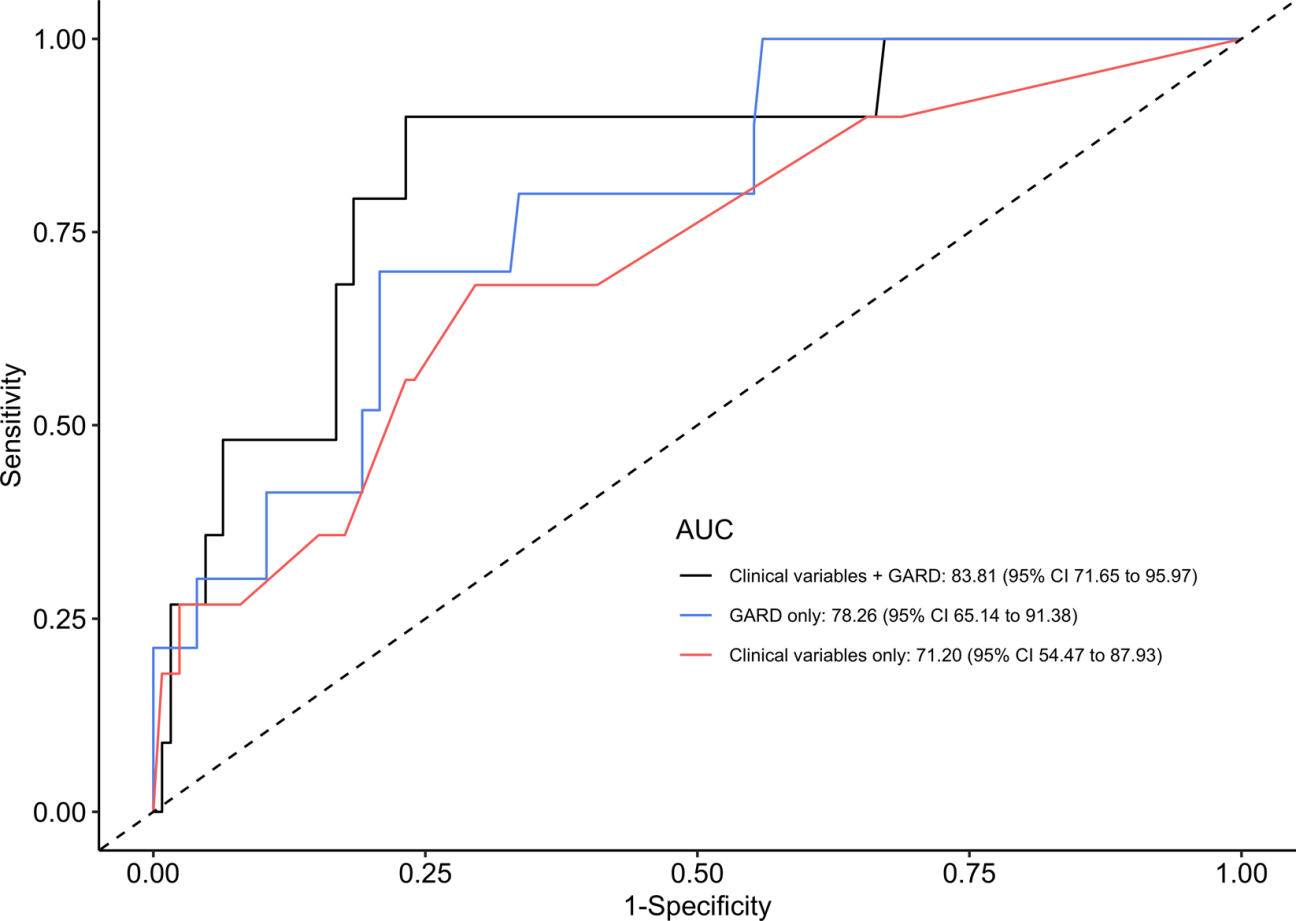
AUC analysis shows GARD outperforms standard clinical variables. In this AUC analysis, we compared Cox regression of clinical variables (red) with GARD alone (blue) and a combined model using all factors (black). The combined model shows dramatic improvement compared with clinical variables alone. This model included all 191 definitive primary RT patients and analyzed outcome at 3 years.

In addition, we developed and evaluated a Cox regression model including the previously known prognostic clinical variables (T stage, N stage, smoking, and ECOG performance status) to determine whether a model including GARD improves overall model performance. As shown in Figure 3, the Cox model including clinical variables achieved an AUC of 71.20, whereas GARD alone achieved a superior AUC (3-year OS) of 78.26. Integrating GARD with the clinical variables improved the prognostic ability of the model with AUC 83.81. We also evaluated the previously developed 3-cluster model (12), which, by itself, achieved an AUC similar to the clinical variable model (AUC = 72.83). However, integration of the 3-cluster information into the GARD plus clinical variable model did not improve the overall prognostic ability, as measured by AUC 83.81. This lack of improvement may be related to the relationship between clusters and GARD values (see Supplemental Figure 3 and Supplemental Table 4).

### GARD predicts that empiric dose de-escalation would result in inferior clinical outcome.

**Figure 4:**
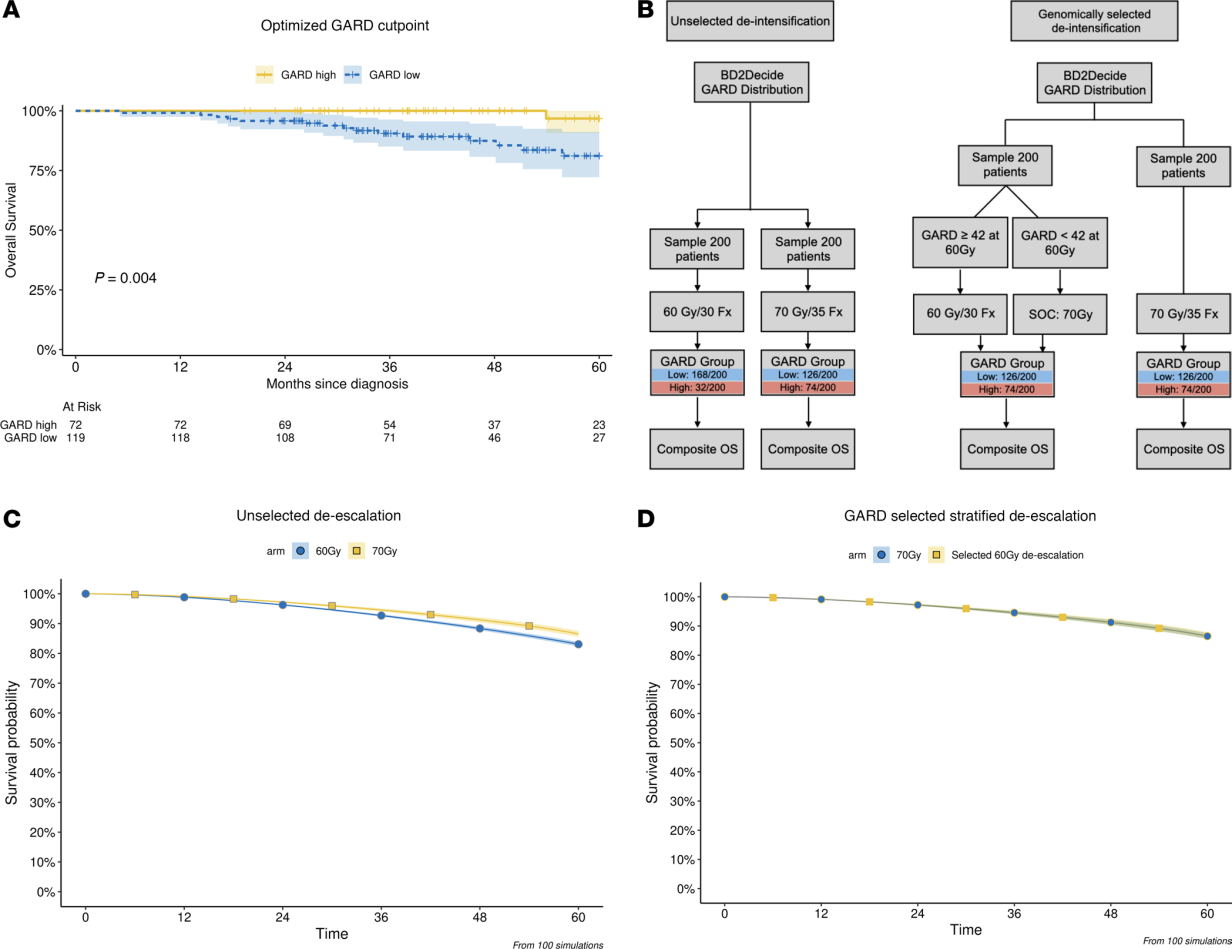
GARD predicts that uniform RT dose de-escalation in HPV^+^ patients would result in an inferior clinical outcome compared with SOC. (**A**) GARD-identified HPV^+^ patient subsets with differential risk of failure. An exploratory analysis identified 1 cut point that grouped patients in 2 risk levels. Patients that achieved the lowest GARD (<42) had a higher risk of failure (3-year OS = 90.5%). Statistical significance was evaluated using a log-rank test from Kaplan-Meier estimates. (**B**) In silico clinical trial designs. Left: Simulated unselected RT dose de-escalation (cisplatin + 60 Gy vs. cisplatin + 70 Gy). We utilized the RSI distribution of the BD2Decide cohort to generate GARD for 400 virtual patients randomized to either 60 or 70 Gy and repeated this 100 times. Right: GARD-based de-escalation. In 1 example of a potential trial, we simulated a GARD-selected trial where only patients with GARD ≥ 42 were eligible for randomization to de-intensification. Fx, fractions. (**C**) Simulation of unselected RT dose de-escalation (cisplatin + 60 Gy) resulted in inferior OS compared with SOC (cisplatin + 70 Gy). The unselected in silico clinical trial predicted that patients treated with RT dose de-escalation experience a statistically significantly worse OS when compared with SOC (3-year OS of 92.7% vs. 94.6%, nonoverlapping CIs). (**D**) Selective de-intensification produced similar OS.

Although HPV^+^ patients have excellent prognosis, the interim analyses of HN005 have emphasized the importance of developing clinical tools to identify patient subsets with differential risk of clinical failure. We hypothesized that GARD could identify a subpopulation of HPV^+^ patients at differential risk of failure that may explain the failure of unselected empiric dose de-escalation as tested in HN005. In addition, understanding the differential risks of failure can lead to a better clinical strategy for dose de-escalation in selected patients. To develop this, we performed an exploratory discrete analysis based on an optimized cut point analysis. Minimizing the log-rank score at 1 discrete value revealed 2 groups with maximally different outcomes (see Supplemental Figure 4). This analysis revealed 1 cut point at GARD < 42 that optimally stratified patients, as shown in Figure 4A.

Patients in the GARD-high group (GARD ≥ 42) had a 3-year OS of 100% (CI: 1–1) compared with 90% (CI: 0.85–0.96) for the GARD-low group (GARD *<* 42). These differences are statistically significant with *P* = 0.0045, though this analysis should be interpreted carefully as it was performed for hypothesis generation and the groups were chosen by maximizing differences post hoc.

One possible explanation for the HN005 results is that empiric dose de-escalation results in a small number of patients falling from the GARD high cohort (GARD ≥ 42) to the GARD-low cohort (GARD *<* 42), leading to an inferior result for empiric dose de-escalation. To test this hypothesis, we performed an in silico clinical trial to evaluate GARD-based predictions of clinical outcome for empiric dose de-escalation to 60 Gy (with concurrent chemotherapy) as in HN005 (see Figure 4B for schema). We found that GARD predicts that empiric (unselected) dose de-escalation would result in an inferior clinical outcome. The predicted 3-year OS for patients modeled at 70 Gy was 94.6% compared with 92.7% for patients modeled at 60 Gy (Figure 4C). Empiric unselected dose de-intensification is predicted to increase the proportion of patients in the GARD-low group while decreasing the proportion of patients in the GARD-high group. The 70 Gy in silico arm had an average of 126 and 74 patients in the low and high GARD groups, while the 60 Gy in silico arm had 168 and 32 patients in those groups.

Next, we determined whether we could use GARD to develop a clinical trial strategy that would predict an equivalent outcome at 70 or 60 Gy. In 1 approach, GARD can identify patients that would remain at or above the GARD-high cut point (≥42) at 70 or 60 Gy. Based on simulations, approximately 16% of the HPV^+^ trial population would be eligible for dose de-escalation in this scenario. It should be noted that this approach excludes GARD-low patients and patients that fall from GARD high to GARD low at 60 Gy. The predicted OS curve for this approach to de-escalation is shown in Figure 4D. The 36-month survival proportion is equivalent (94.6%) in both arms of this simulated trial.

**Figure 5:**
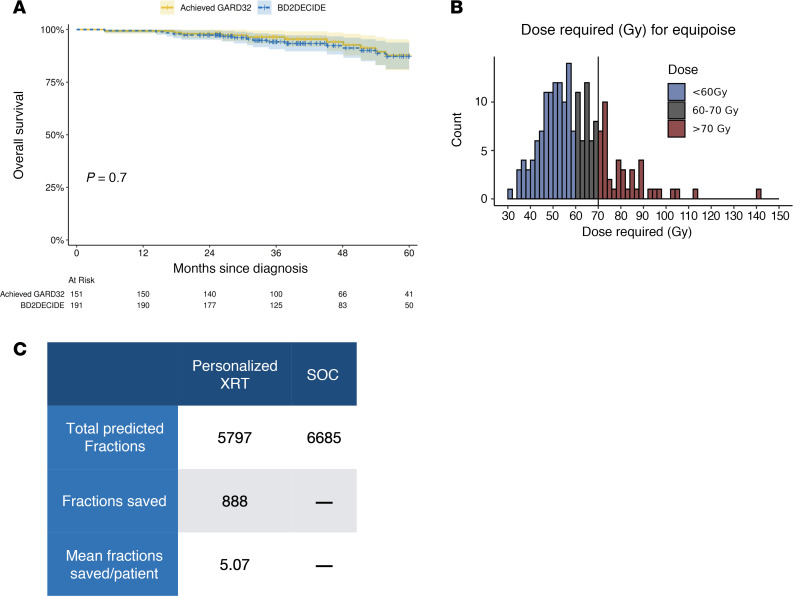
GARD-based opportunities for equipoise targeted RT dose reduction: maintaining equivalent outcomes to SOC. (**A**) GARD targeted equipoise RT dose reduction. Kaplan-Meier curves show that patients in the BD2Decide cohort that had a GARD of at least 32 achieved isocurative outcomes compared with the unselected cohort. Each patient could then have a prescription RT dose to match the target GARD (at least 32). Comparing this to SOC provided equivalent outcomes. Statistical significance was evaluated using a log-rank test from Kaplan-Meier estimates. (**B**) A histogram depicting the difference between the dose predicted to be required for each patient compared with the dose delivered. Red indicates patients that were underdosed (39 of 175), offering opportunities to increase oncologic outcomes, and blue indicates patients that were overdosed, suggesting opportunities to decrease toxicity. (**C**) Calculating the difference between fractions delivered in SOC compared with the number predicted on a per patient basis revealed a large potential for toxicity reduction at the population level. This averaged approximately 5 fractions (1 week of radiotherapy) per patient, but with large heterogeneity. XRT, radiation therapy.

Another way to think about dose de-escalation is to ask the question: Can GARD identify a personalized target dose with the goal of maintaining current outcomes? This is fundamentally different than our previous approach, which asked if we could use GARD to select patients for stratified de-escalation to standard dose levels. In this approach, we instead asked what GARD cut point would provide equipoise to current SOC? Analyzing our cohort through this lens, we found that a substantially lower GARD cut point equal or higher than 32 (see Supplemental Figure 5) would provide outcomes in line with current SOC. Figure 5A shows the outcome of patients that achieved GARD 32 compared with unselected patients in the BD2Decide cohort. As shown, the patients that achieved a GARD of at least 32 had the same OS as the whole unselected cohort, thus achieving equipoise with current SOC in unselected patients.

In a trial designed like this then, each patient would be assessed for their RSI and then a physical radiation dose would be calculated such that they would achieve a prescribed GARD of at least 32. While exploratory and nonstandard, this analysis of a genomic prescription paradigm offers us a window into the future where dose is truly personalized, allowing exactly enough radiation to be delivered for tumor cure, minimizing toxicity. In Figure 5B shows the minimum dose required for each patient in the BD2Decide cohort to achieve a GARD of at least 32. Interestingly, the average dose needed aligns well with our clinical intuition — approximately 60 Gy — but with large heterogeneity across individual patients. Of note as well is the large number of patients (22.3% in this cohort, 39 of 175) who we predict require between 60 and 70 Gy, revealing which patients would have inferior outcomes when de-escalated to 60 Gy.

This analysis also suggests that on average the toxicity (financial and clinical) of nearly 5 fractions/patient can be spared while maintaining similar outcomes (Figure 5C). However, the potential toxicity reduction for each patient is variable, with some patients predicted to only require 30 Gy, while a small minority may require higher doses than standard. Of note, there was a peak in the distribution between 60 and 70 Gy, meaning that as we reduced dose from 70 to 60 Gy without genomic guidance, we underdosed a substantial portion of patients, worsening our outcomes. This stands in contrast to our findings in non-small-cell lung cancer (15), where the dose escalation from 60 to 74 Gy (as in RTOG 0617) spanned a valley in the distribution, meaning that the escalation resulted in very few patients having a treatment benefit, while all received the increased toxicity.

## Discussion

The development of prognostic and predictive models to more accurately prescribe therapies is a central goal of personalized oncology. In this paper, we show that GARD, a previously described predictive model of the treatment effect of RT, is associated with OS in HPV^+^ oropharyngeal cancer patients treated with RT as a continuous and optimized dichotomous variable. Furthermore, using time-dependent receiver operating characteristic (ROC) analysis, we show that GARD outperforms standard clinical variables for prediction of OS of these patients and that combining genomics (GARD) with clinical factors provides a superior model to either alone (16). Finally, we show that GARD provides an opportunity to depart from the uniform RT dosing strategies that are hindering the improvement in outcomes for our patients. Instead of searching iteratively for subgroups that can be uniformly dose optimized, we propose a strategy where each patient can have their dose (and plan) optimized based on their individual biology. Our analysis is limited by several factors, including that it focused only on OS (recurrence information was not provided) and the patients included were treated in a relatively long span of time (2008–2017), during which there was significant improvement in RT techniques. However, to this point almost all patients (99%) were treated with volumetric arc therapy or intensity modulated radiation therapy technique.

With the aim of breaking the mold in radiation oncology trial design, we propose 2 GARD-based strategies to treatment optimization. The first prioritizes individual oncologic outcome for every patient, without considering concomitant toxicities at the population level. We found that personalizing RT dose to achieve GARD ≥ 42 maximized oncologic outcome in this dataset. However, only 16% of patients achieved GARD ≥ 42 at 60 Gy, limiting this approach if the goal is to reduce toxicity. In the second approach, we define a risk of failure that matches the risk of an unselected population treated at 70 Gy, creating a greater opportunity to reduce RT dose and toxicity risk. If we based the GARD model on these parameters, personalizing RT dose to achieve GARD of at least 32 would achieve equipoise with current population-level oncologic outcomes while reducing RT dose, and therefore toxicity, to the majority of patients.

In this approach, a larger proportion of patients became candidates for de-escalation (77.7%), while a small fraction were identified for potential escalation (22.3%). This strategy matches current clinical outcomes but delivers an average of 5 fewer fractions per patient and thus achieves the intended aim of HN005 of equipoise at an average dose of 60 Gy. However, the key difference is that the personalized dose for each patient is not 60 Gy, but instead, like most polygenic biological traits, a wide range (between 31 and 113 Gy). While the majority of patients require lower doses, a small minority may need dose intensification or a more effective radiosensitization strategy with concurrent chemotherapy. Further work on chemotherapy sensitivity (17) and balancing toxicity with tumor control (15) could also inform future iterations of these trials, most of which include cisplatin as a radiosensitizer (18).

In a previous study, we introduced the concept of the toxicity cost of RT dose personalization; the optimization of RT dose can result in increased or reduced risk of normal tissue toxicity (15). This toxicity cost/gain is also different for each patient. In this analysis, GARD offers a genomics-based strategy that achieves clinical equipoise while decreasing average dose by 5 fractions/patient. Thus, by accounting for biological heterogeneity, GARD-based RT prescription provides critical data that may improve the therapeutic ratio of RT, maximizing clinical outcome at the lowest possible toxicity risk.

In conclusion, we demonstrate that GARD outperforms standard clinical variables as a prognostic biomarker in HPV^+^ OPSCC patients and provides a biology-based approach to radiotherapy dose personalization. GARD predicts that while the majority of patients require lower doses than SOC, a small minority of resistant patients still require 70 Gy or potentially a higher dose to maintain an excellent clinical outcome. Thus, an unselected uniform RT dose reduction approach fails because it does not recognize biological heterogeneity in radiosensitivity. To fully realize the clinical potential of radiotherapy in HPV^+^ oropharynx cancer patients, we must integrate the assessment of biological heterogeneity into our treatment strategy. Integrating GARD into the clinical evaluation of HPV^+^ oropharynx patients is a step in that direction, bringing radiation oncology 1 step closer to truly personalized medicine.

## Methods

### Sex as a biological variable.

Sex was not considered as a biological variable in this study.

### Patient cohort.

Patients in this analysis were part of the BD2Decide Project (ClinicalTrials.gov NCT02832102), a collaboration of 6 European centers to develop a clinico-genomic database of head and neck cancer patients. The details of this project have been previously described (10). Briefly, BD2Decide enrolled 1,537 patients (*n* = 1,086 retrospectively and *n* = 451 prospectively) with loco-regional advanced head and neck cancer (stages III–IVa and IVb, AJCC Seventh Edition, or Stage I–III, AJCC Eighth Edition) treated with curative intent, including 377 patients with HPV^+^ oropharyngeal cancer. Of these, 286 patients had gene expression profiling available (Gene Expression Omnibus [GEO] data GSE163173).

As shown in Supplemental Figure 1, after excluding patients that were HPV DNA^–^ (*n* = 14), patients with locally advanced disease that underwent single-modality treatment (*n* = 37), patients treated with surgery alone (no GARD could be calculated) (*n* = 1), and patients with postoperative RT (*n* = 43), a final study population of 191 patients remained. A total of 172 of 191 patients were treated concurrent chemotherapy and RT, while 19 of 191 received RT alone. The study was approved by IRBs of each of the participating institutions and when possible patients consented to enrollment, or a waiver for consent was approved. Patients were treated between 2008 and 2017, and follow-up closed in September 2019 (10).

HPV testing was performed with p16 IHC and confirmed by HPV DNA testing following positive staining (10). In total, 191 patients received definitive RT primary treatment. Fifteen RT dose fractionations were prescribed for definitive RT cases, with the 2 most common being 70 Gy in 35 fractions or 69.96 Gy in 33 fractions. Median RT dose was 70 Gy (range = 51–74; IQR = 69.96–70.0) for definitive RT cases. The median follow-up was 43.95 months (IQR = 33.0–60.7)

### Bioinformatics.

Quarto markdown documents and data preprocessing scripts are available at https://github.com/steveneschrich/GARDHNC (branch: main; commit: 3eddd0f). A full package list with versions is available as a ‘renv’ lock file at the site above. Graphs were generated using ggplot2 and ggpubr R packages.

All patient tumors previously underwent gene expression profiling using Affymetrix Clariom D from formalin-fixed samples and are available from the GEO database (GEO GSE163173). Raw CEL files were processed and normalized using Affymetrix sst-rma in the Expression Console (Thermo Fisher Scientific). RSI values were then generated using a 10-gene signature as previously described (12, 19) and as implemented in the R package hacksig (20). RSIs have been previously clinically validated in multiple cohorts (13, 14, 21–27). A patient-specific genomic parameter, α*_g_*, was subsequently calculated using the linear quadratic model to estimate patient radiosensitivity, the derivation of which we have previously described, yielding the relation
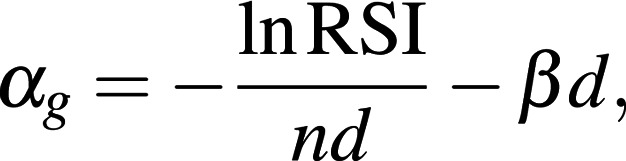


where dose *d* is 2 Gy, and the number of fractions (*n*) is 1, as this moves from a genomic measure to the familiar surviving fraction after 2 Gy. We further make the simplifying assumption that β is a constant at 0.05/Gy^2^. This genomic parameter, α*_g_*, is then used together with each patient’s specific radiation dose and fractionation to calculate their clinical GARD value (GARD*_c_*),
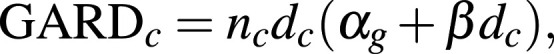


where *n_c_* is the number of fractions, and *d_c_* is the dose per fraction per the clinically delivered radiation plan to each patient. These values were calculated without information about clinical outcome using the previously described model. Details of the derivation of α*_g_* and GARD*_c_* can be found in Scott et al. (15).

OS comparisons were performed using the survival, gtsummary, and rms packages. Cox proportional hazards regression from the R package rms was used to assess the association between GARD as a continuous variable and OS. Multivariable Cox regression analysis was performed using survival and gtsummary packages. Survival curves were generated using the R package survminer. Discrete analyses with log-rank statistics were performed for hypothesis generation using an algorithm to minimize the log-rank statistic to derive optimal groups in 2 dose levels. The R packages survminer and maxstat were used for this analysis.

OS was defined as the time between primary diagnosis and death or last follow-up. Follow-up was censored at 60 months if no event occurred prior. After performing standard analyses to determine the association of GARD with outcome, we incorporated GARD with known prognostic clinical variables including stage, smoking pack years, and ECOG performance status to determine whether GARD improved prognostic performance. In addition, we also integrated the 3-cluster gene expression model (12) following standard methods (28). The Cox proportional hazard models generated by the rms R package were evaluated by comparison of time-dependent ROC curve analysis with the timeROC package in R (29).

### In silico clinical trial simulations.

We first performed an in silico trial to test uniform RT dose de-intensification (from 70 to 60 Gy) in this cohort of HPV^+^ oropharyngeal cancer patients. Virtual patients were generated by random sampling from an estimated density distribution derived from the measured RSI distribution from the BD2Decide cohort. Specifically, sampling (with replacement) from the existing RSI values ensured sampling according to the initial empirical probability distribution. A Gaussian kernel (with estimated bandwidth via nrd0) was sampled and added to each RSI value, ensuring a similar distribution. Thus, each cohort of virtual patients would have a similar RSI distribution as BD2Decide. Virtual patients were generated for each trial arm (*n* = 200), with 400 total patients per trial, for standard chemo-RT (70 Gy) versus de-escalated chemo-RT (60 Gy). The in silico trial was repeated 100 times. Outcomes were estimated based on a Weibull distribution of the original outcomes evaluated at discrete time points, with GARD-high and -low populations modeled independently. The combination of low/high GARD population distributions yielded the overall cohort outcome estimates. GARD was calculated for each virtual patient, and an OS curve was predicted based on the GARD level achieved, by weighted average of the optimized 2-dose GARD level approach (GARD < 42 = low and GARD ≥ 42 = high). This cut point was identified as previously described (see Supplemental Figure 4).

To determine whether GARD could identify a successful de-intensification strategy, we performed a second 2-arm in silico trial in which only a GARD-identified subset of patients were de-intensified in 1 arm. The control arm was treated uniformly at 70 Gy, as above. In the in silico experimental arm, only GARD-high patients (GARD ≥ 42) who remained in the same risk group after de-escalation to 60 Gy were de-intensified. All other patients remained at 70 Gy. In other words, GARD-low (high-risk) patients were treated at 70 Gy, and GARD-high patients were de-intensified to 60 Gy unless this changed their risk category.

As a final exploratory analysis, we sought to determine what a purely personalized approach, with a GARD target chosen to provide an overall outcome equivalent to our current SOC, would reveal. In this personalized iso-curative dosing strategy, we compared a SOC arm (70 Gy in 35 fractions) with a target GARD identified to provide equipoise to modern outcomes (in this case, GARD = 32) (see Supplemental Figure 5). We then calculated the total difference in dose predicted to provide equipoise across the population and the differential in cost to provide this at the population level.

### Statistics.

All analysis was performed in R version 4.4.2 and associated packages unless otherwise noted. For survival analysis tests, Cox regression and log-rank tests were used; differences in continuous values by category were assessed by Kruskal-Wallis test. *P* values of less than 0.05 were considered significant.

### Study approval.

This research study is associated with a published trial (ClinicalTrials.gov NCT02832102) (10). No individual IRB approval was needed.

### Data availability.

The clinical and molecular data used in this study are owned by the BD2Decide consortium and the participating clinical centers. Due to ethical, legal, and privacy restrictions under the European General Data Protection Regulation, most of the data cannot be made publicly available. However, deidentified data may be made available from the corresponding author upon reasonable and motivated request, subject to approval by the BD2Decide data governance committee and the relevant institutional ethics bodies. In addition, MIAME-compliant gene expression data have been anonymized and deposited in the GEO (GSE163173). Values for the data presented in this work are available in the Supporting Data Values file.

## Author contributions

EH, SAE, MWK, JFTR, and JGS conducted experiments, analyzed data, and wrote the manuscript. LDC and LL acquired data, analyzed data, and wrote the manuscript. FH, RHB, KS, and TP acquired data and wrote the manuscript. S Cavalieri, GS, KY, JAS, SN, S Campbell, NW, TC, JM, NS, SK, JB, and JJC analyzed data and wrote the manuscript.

## Supplementary Material

Supplemental data

Supporting data values

## Figures and Tables

**Table 1:**
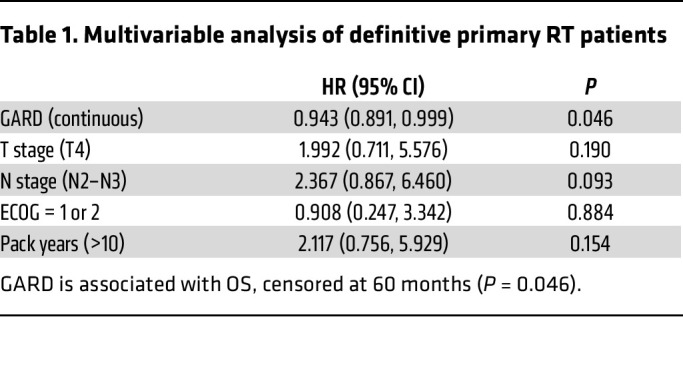
Multivariable analysis of definitive primary RT patients

## Version 1 09/25/2025

In-Press Preview

## Version 2 10/01/2025

Electronic publication
