# Supplementary material for: Personalized treatment in HPV+ oropharynx cancer using genomic adjusted radiation dose
Source: J Clin Invest. 2025 Sep 25;135(19):e194073. doi: 10.1172/JCI194073 (PMC12483556; doi:10.1172/JCI194073)
Supplement: Supplemental data [file jci-135-194073-s149.pdf]

Supplemental Information

Code

Statistical analyses were conducted using R v4.4.2 and associated packages. All quarto/R scripts and data preprocessing R scripts are available at https://github.com/steveneschrich/GARDHNC.

Conflict of Interest

SAE and JTR hold patents and are co-inventors, co-founders and stock holders of Cvergenx, Inc. SAE is board member of Cvergenx, Inc. JGS holds patents and is a stock holder of Cvergenx, Inc.

Study Cohort

Patients from this analysis were part of the BD2Decide project. HPV-positive oropharyngeal cancer patients (N=234) with primary definitive RT and evaluable RSI/GARD (Supplemental Figure 1) were identified and characterized (Supplemental Table 1). work.

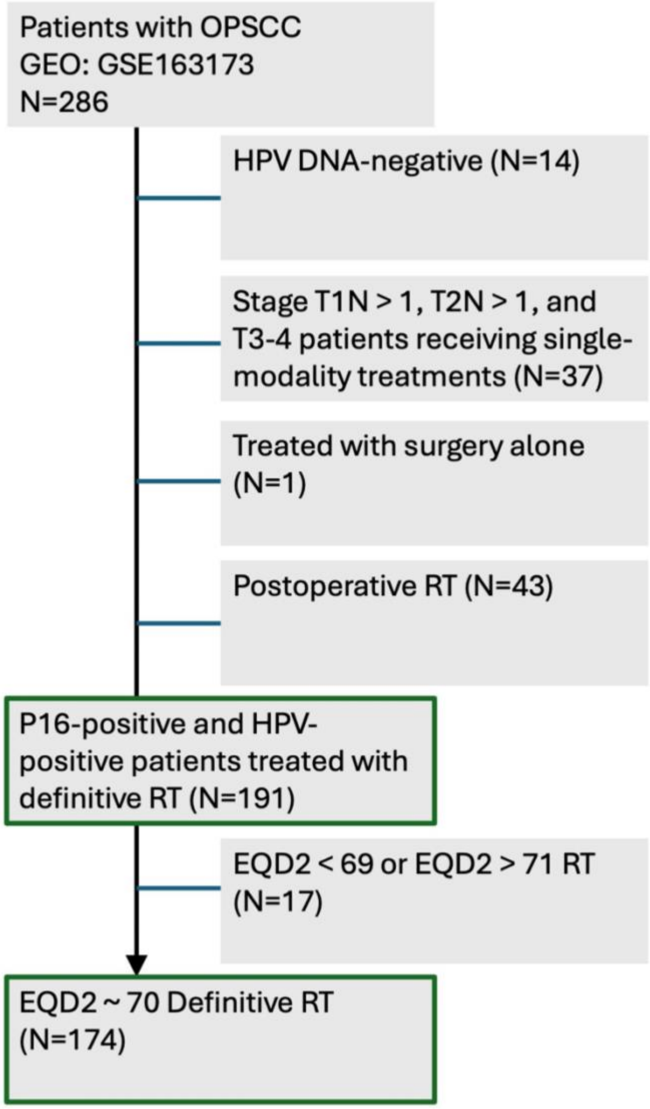

Supplemental Figure 1. CONSORT diagram for study cohort. Green Boxes indicate cohorts used within this work

Characteristic	N = 191
AJCC8, n (%)	
I	87 (46)
II	49 (26)
III	55 (29)
ECOG, n (%)	
0	158 (83)
1	32 (17)
2	1 (0.5)
Smoking Pack Years, Median (IQR)	8 (0 – 30)
AJCC8 T stage, n (%)	
T1	38 (20)
T2	57 (30)
T3	45 (24)
T4	51 (27)
AJCC8 N stage, n (%)	
N0	6 (3.1)
N1	143 (75)
N2	34 (18)
N3	8 (4.2)
status, n (%)	
Alive	172 (90)
Dead	19 (9.9)

Supplemental Table 1. Characteristics of identified BD2DECIDE patient cohort (N=191). Counts are provided in parentheses except for Smoking Pack Years, which is reported as median and interquartile range (IQR)

Characteristic	HR	95% CI	p-value
stage	2.48	0.80, 7.70	0.115
Tstage	2.31	0.86, 6.22	0.096
Nstage	2.72	1.01, 7.32	0.048
ECOG	1.32	0.38, 4.64	0.664
pack_years	2.06	0.75, 5.66	0.163
StandardRTDose	0.40	0.11, 1.41	0.155

Supplemental Table 2. Univariate overall survival associations of identified BD2DECIDE patient cohort (N=191; 16 events). Dichotomized clinical variables are assessed for relationship to overall survival using univariate Cox regression. Abbreviations: CI = Confidence Interval, HR = Hazard Ratio.

RSI and GARD Distributions

Distributions of RSI and GARD grouped by AJCC8 stage are shown in Supplemental Figure 2.

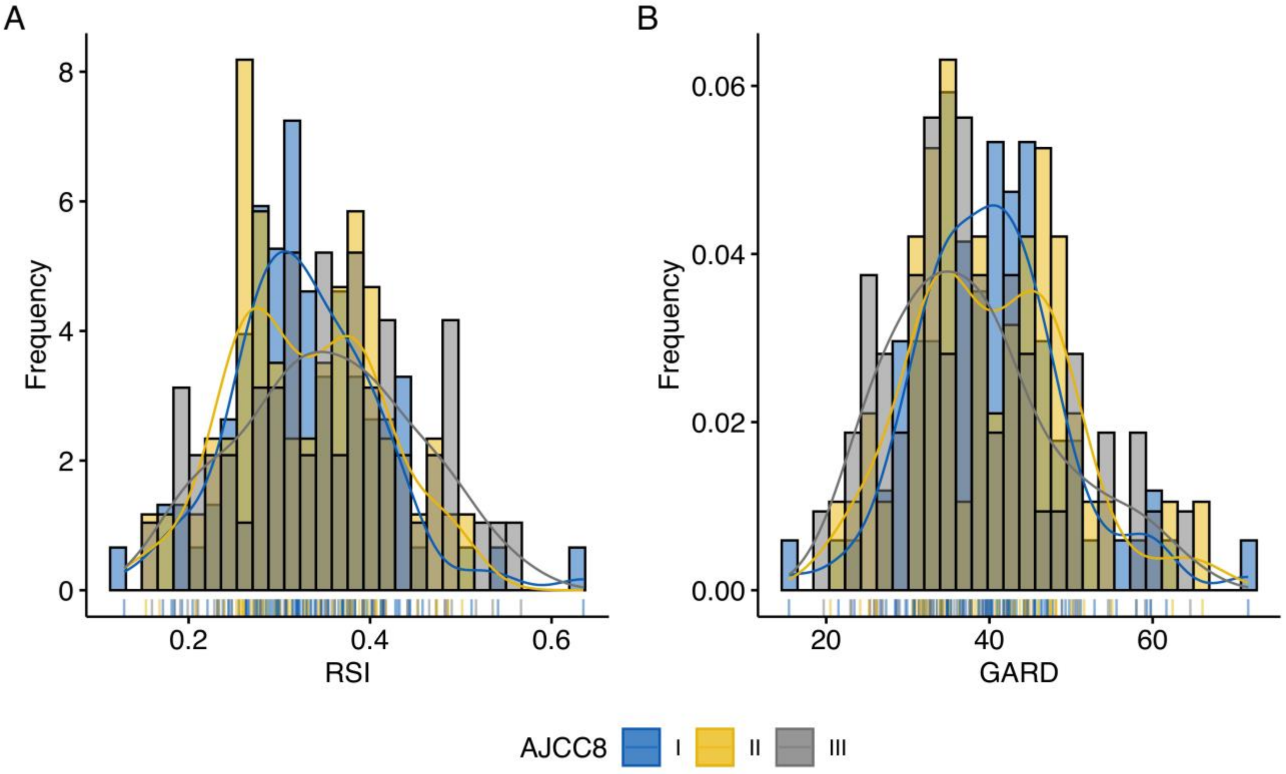

Supplemental Figure 2. The distributions of RSI and GARD for each AJCC8 stage are similar.

Kruskal-Wallis tests revealed no significant differences among these distributions (by stage), as shown in Supplemental Table 3.

	Stage	N	Median (IQR)	p
RSI	I	87	0.32 (0.28 – 0.38)	0.23
	II	49	0.33 (0.26 – 0.39)	
	III	55	0.35 (0.28 – 0.41)	
GARD	I	87	40 (34 – 46)	0.25
	II	49	39 (34 – 47)	
	III	55	37 (31 – 45)	

Supplemental Table 3. The p-values from the Krusal-Wallis rank sum test for differences in RSI/GARD distributions across stage. Median (IQR, interquartile range) values shown.

AUCs for various predictors

Comparison of the 3 most significant predictors individually via ROC analysis showed the greatest AUC for GARD alone (78.26), while the model combining GARD, T stage, N stage, smoking and ECOG status produced the highest AUC (83.81); values are listed in Supplemental Table 4. Of note, if RSI is compared here with the same cohort, a similar score to GARD is achieved of 77.68 (95% CI: 65.13 to 90.23) as the dose range in this cohort is narrow.

	3-yr AUC (95% CI)
Clinical	71.20 (54.47, 87.93)
Clusters	72.83 (59.01, 86.65)
GARD	78.26 (65.14, 91.38)
Clinical + GARD	83.81 (71.65, 95.97)
Clinical + GARD + Clusters	83.81 (71.65, 95.97)

Supplemental Table 4. The time-dependent AUC and 95% CI at 3 years are shown for the predictors individually as well as for the combined nomogram.

GARD vs Clusters

The prognostic gene expression clusters defined by Locati (30) and Cavalieri (12) are related to the GARD levels (particularly for patients with low GARD/High Risk), see Supplemental Figure 3. Kruskal-Wallis tests revealed significant differences in GARD among these clusters. Note that in the original publications the clusters were reported as RSI distributions, which we have transformed here into GARD using Eq 1 and 2. We have kept the cluster numbers as per the original publication.

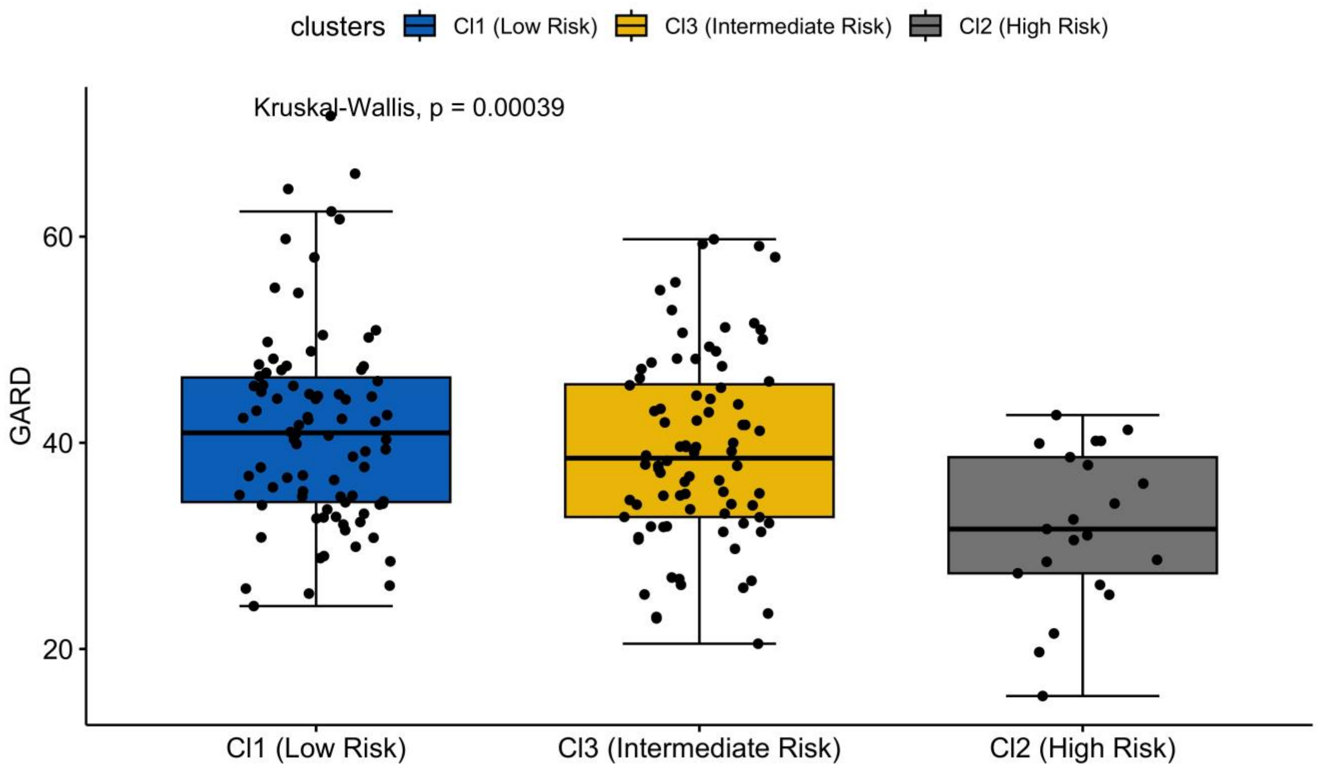

Supplemental Figure 3. GARD values correlate with risk groups as calculated by gene expressions clusters. Boxplot of GARD values across the three defined cluster subtypes. GARD was calculated as described in the Methods for each patient. Statistical significance was evaluated using a Kruskal-Wallis test.

Calculating GARD thresholds

In order to find GARD cutpoints for our various *in silico* clinical trials, we performed cutpoint analyses with varying endpoints. First, to optimize outcome, we searched for the GARD threshold which optimally separated the groups by OS. In Supplemental Figure 4, we find a cutpoint of GARD 41.97 (42) optimally separates the groups.

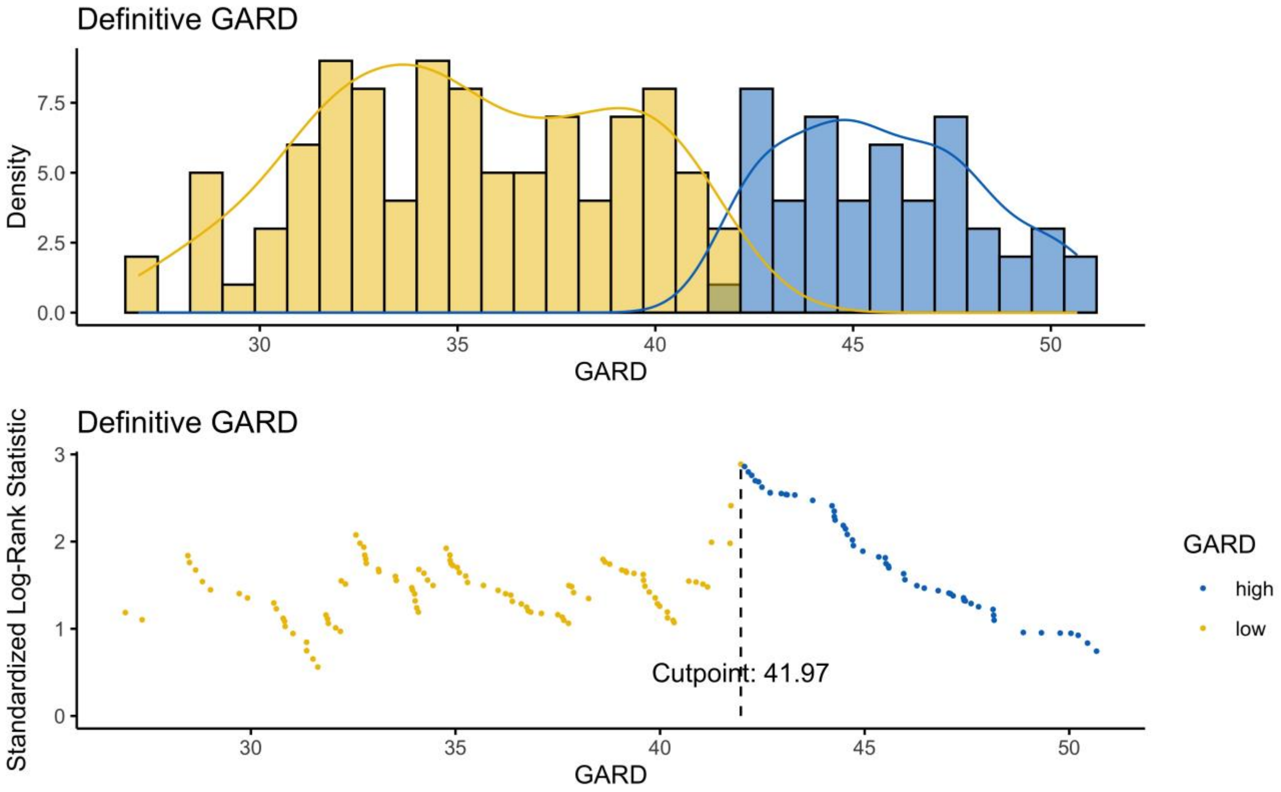

Supplemental Figure 4. Identification of an optimal GARD threshold for outcome using survminer package and chisq statistic optimization.

We subsequently asked which GARD cutpoint would, at the population level, yield equipoise to the SOC, 70 Gy treatment. In Supplemental Figure 5, we find that a GARD cutpoint of 32 yields 97% OS at 3 years.

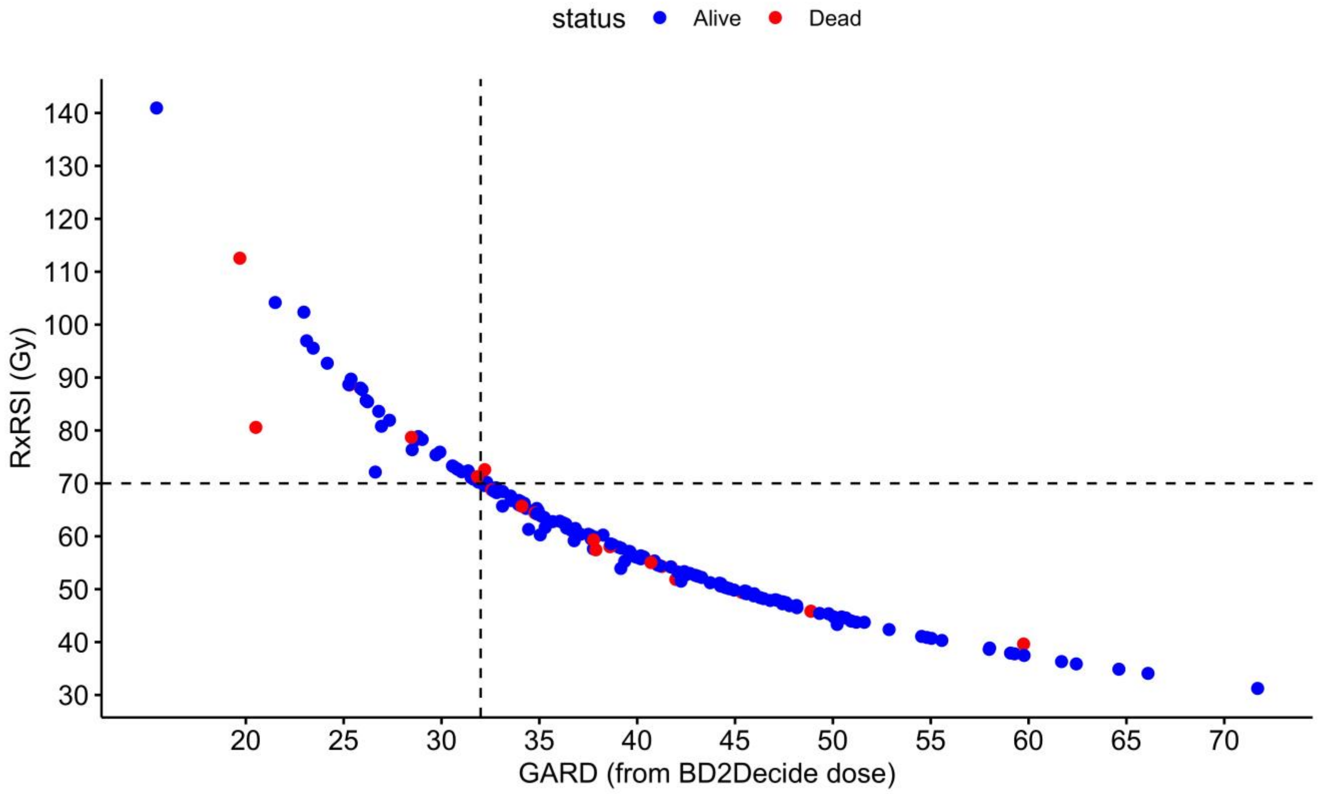

Supplemental Figure 5. Identification of a GARD threshold for outcome to match SOC.
